# Nasal schwannoma

**DOI:** 10.1016/S1808-8694(15)31031-4

**Published:** 2015-10-19

**Authors:** Giulliano Enrico Ruschi e Luchi, Marcos Ribeiro Magalhães, Soraia Maria Lanzelotti, José Jarjura Jorge Júnior, Eduardo Augusto Santana Ferreira de Mendonça, Sandra Lira B. Magalhães

**Affiliations:** a MD. Otorhinolaryngologist. MS in Otorhinolaryngology - Medical Sciences School - Santa Casa de São Paulo.; b MS in Head and Neck Surgery - Hospital Heliópolis.; c MD.; d PhD. Professor of Otorhinolaryngology - Medical Sciences School - Pontifícia Universidade Católica de São Paulo - PUC-SP.; e MD. Otorhinolaryngologist.; f MD. Otorhinolaryngologist. Pontifícia Universidade Católica de São Paulo - PUC-SP.

**Keywords:** epistaxis, nasal obstruction, nasal schwannoma, nasal tumor

## INTRODUCTION

Schwannomas are most commonly located on the VIII CN(80%). They are more rarely found in the nasal region (4%). On the nose, they may rise from the autonomous nervous fibers or from branches of the V CN. Malignant transformation is rare (2%). According to Hillstrom^1^, electron microscopy and immunohistochemistry help in differentiating this tumor from neurofibromas, the latter with more common malignant transformation (12%)^1^, ^2^.

Nasal schwannomas may present progressive unilateral nasal obstruction, with or without epistaxis, hyposmia and headaches. On physical examination we usually only see a grayish mass in the nasal cavity. It may resemble a vegetative lesion or polyp, much vascularized and bleeding excessively. Nasofibroscopy and CT scan are necessary in order to assess tumor extension and surgical approach. Histopathology provides the final diagnosis. Treatment is based on total tumor excision. Pre-operative biopsy helps in the differential diagnosis of other nasal-born tumors.

**Figure 1:**
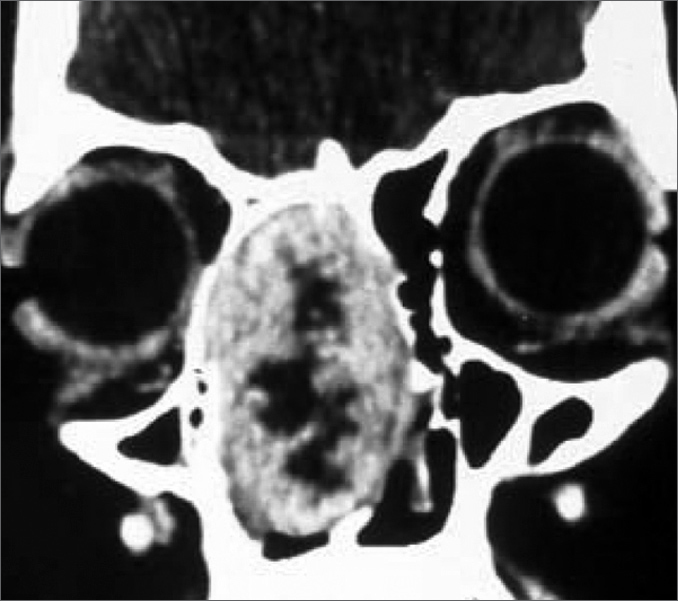
Coronal CT scan image showing a tumor in the right nasal cavity, with intact intracranial and orbital limits.

## CASE PRESENTATION

18 year old woman with volume increase in the nasal pyramid to the right side and recurrent epistaxis that ended spontaneously for 6 months, nasal obstruction and hyposmia. Anterior rhinoscopy showed a purple-like soft mass, painless to palpation, totally occluding the right nasal cavity. Paranasal cavities CT scan showed a highly vascularized lesion on the anterior region of the nasal cavity that did not extend to the nasopharynx. Biopsy was carried out in an outpatient basis and the pathology exam diagnosed inflammatory polyp with metaplastic cover. A lateral rhinotomy was carried out with tumor exeresis and cauterization of the resection bed. Histopathology exam of the surgical specimen proved it to be a benign nasal schwannoma. After 18 months there was no evidence of local recurrence.

## DISCUSSION

There are about 70 reports of nasal schwannomas in the literature. Most in adults between forty and sixty years of life, while our patient is 18 years old. According to Hasegawa^3^, the age of patients with this type of ailment varied between 12 and 76 years.^1^, ^2^, ^3^ In Leakos's^4^ report, the patient presented with nasal obstruction, anosmia and nasal pyramid deformity, but not epistaxis. The cases reported by Lacosta^5^ and Alessandrini^6^ complained only of nasal obstruction. Hasegawa^3^ revised 6 cases and concluded that the most common symptom is nasal obstruction, but it also included epistaxis, as it happened to our patient.^3^, ^4^, ^5^, ^6^ Most of the cases reported showed grayish tumors seen at anterior rhinoscopies, however the tumor in our case was purple-like. More commonly, CT scan showed tumor invasion of the paranasal cavities, nasopharynx and intracranial region, however in our case the tumor was limited to the nasal cavity. Surgical approach through lateral rhinotomy, as we did, is the preferred technique by most of the authors consulted, however there are also reports of endoscopic removal of such tumor.

## FINAL COMMENTS

Since it is a rare tumor, just like in literature reports, diagnosis suspicion of nasal schwannoma was not considered at first, such diagnosis was confirmed by histology.
